# Single-Particle Tracking via Direct Labeling of the Viral Lipid Envelope with Cholesterol-DNA-Functionalized Quantum Dots

**DOI:** 10.3390/v18101072

**Published:** 2026-09-28

**Authors:** Wen Yin, Xiaoxu Guo, Jie Zhou, Guobin Mao, Yingxin Ma

**Affiliations:** 1 State Key Laboratory of Biocatalysis and Enzyme Engineering, Hubei Key Laboratory of Industrial Biotechnology, School of Life Sciences, Hubei University, Wuhan 430062, China; 2 Materials Artificial Intelligence Center, Shenzhen Institute of Advanced Technology, Chinese Academy of Sciences, Shenzhen 518055, China

**Keywords:** quantum dots, enveloped virus labeling, cholesterol-DNA functionalization, single-virus imaging, SARS-CoV-2 spike-pseudotyped virions

## Abstract

Quantum dots (QDs) provide rapid, continuous, and prolonged imaging of individual molecules and viruses, offering more comprehensive dynamic data than traditional fluorophores. However, their application in visualizing viral infections is limited, mostly due to the absence of a mild, efficient, and applicable labeling technique. This study presents a simple and gentle technique for labeling enveloped viruses with QDs, requiring only a single brief incubation phase. This method relies on the inherent attraction of the viral lipid envelope to cholesterol (chol), which is shown on Cadmium telluride (CdTe) QDs via Chol-DNA functionalization. The QDs were synthesized in a single step by the coordination of Cd^2+^ ions with phosphorothioate-modified DNA strands, maintaining high fluorescence efficiency and small particle size while enabling DNA modification. This labeling technique attains great efficiency in about one hour while maintaining viral structural integrity and infectivity. Employing this lipid-specific technique, this study established dual-color fluorescent SARS-CoV-2 spike-pseudotyped virions by labeling the envelope with QDs and the nucleic acid with Ru[(phen)_2_(dppz)]^2+^, facilitating real-time monitoring and analysis of infection dynamics. The established lipid-specific QD labeling technique provides an efficient and versatile tool for visually monitoring viruses and analyzing their infection paths, thus promoting broader utilization of single-virus identification and improving comprehension of complex infection mechanisms.

## 1. Introduction

Single-particle tracking is an effective method for examining dynamic molecular processes within living cells, especially for virus–cell interactions [1,2]. A basic requirement for performing this method is the fluorescence labeling of target items. In the last ten years, numerous fluorescent tags, such as organic dyes, fluorescent proteins, Ru[(phen)_2_(dppz)]^2+^, and quantum dots (QDs), have been utilized to mark specific molecules or viruses [3,4,5,6,7,8]. Among these, QDs are differentiated by their distinctive optical features, rendering them especially beneficial for observing individual molecules or viruses [9,10]. QD-labeled individual molecules or viruses can be tracked rapidly and continuously over prolonged durations, enabling the analysis of interactions with various other molecules [11,12,13]. This offers more comprehensive and complex insights into cellular dynamics than labeling with alternative fluorophores. However, the lack of a robust viral labeling mechanism limits their use in virus tracking, thus inhibiting the wider application of single-virus tracking techniques [14,15].

To address this methodological gap, various methods for labeling viruses with QDs have been developed, which can be categorized into three main types. The first group depends on the chemical attachment of QDs, either directly or indirectly, to the amino groups found on viral proteins. For example, direct conjugation by linking viral-NH_2_ to carboxylated QD or indirect attachment by biotin-streptavidin binding, facilitated by the interaction of virus-NH_2_-biotin with streptavidin-QD [16,17,18,19]. This method is effective for both enveloped and non-enveloped viruses; however, it may alter viral protein structure and compromise biological activity [20,21,22,23]. The second category utilizes genetic engineering to integrate specific reactive motifs into viral proteins, which are subsequently coupled to complementary functionalized QDs [24,25]. This technique often yields substantial labeling efficiency; however, complexity and prolonged procedures frequently inhibit practical implementation [26]. A third technique alters the viral membrane indirectly by genetically modifying host cells to display reactive groups. Viruses generated in these cells obtain altered membranes that can subsequently be labeled with QDs [27,28,29]. Despite its efficiency, the efficacy of this approach varies significantly based on the specific underlying virus–host system, limiting its generalizability. To overcome these limitations, Zhang et al. proposed a more efficient alternative [30]. This method integrates biotinylated lipids into the viral envelope, then detects them using streptavidin-conjugated QDs. This technique increases labeling efficiency while maintaining viral protein integrity, exerting minimal influence on infectivity. However, its dependence on various preparatory steps continues to involve prolonged processing time and operational complexity.

**Scheme 1:**
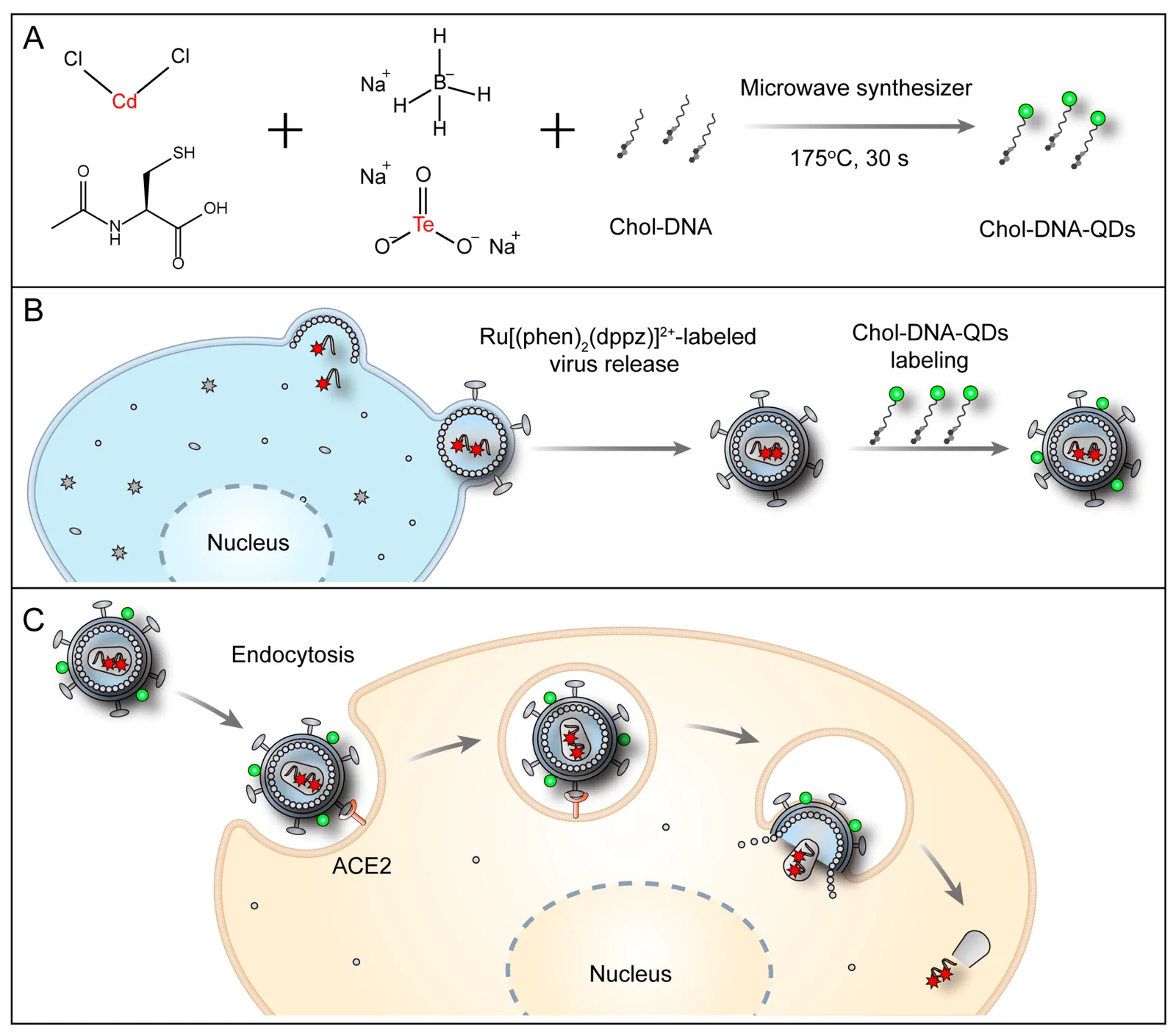
(**A**) Synthetic route of Chol-DNA-functionalized QDs. (**B**) Production of dual-color fluorescent SARS-CoV-2 spike-pseudotyped virions. Virions with Ru[(phen)_2_(dppz)]^2+^-labeled RNA are released from producer cells. Chol-DNA-functionalized CdTe QDs are then attached to the viral envelope to obtain dual-fluorescent virions. (**C**) The dynamic process of viral infection.

This study aims to develop a convenient and efficient strategy for labeling enveloped viruses with QDs while maintaining viral proteins in their original conformation (Scheme 1). We developed a simple yet efficient method to label viral lipid membranes by utilizing the interaction between viral lipids and cholesterol in cholesterol (chol)-DNA-functionalized Cadmium telluride (CdTe) QDs. A one-pot synthesis technique was used to produce Chol-DNA-functionalized QDs, utilizing the coordination between Cd^2+^ ions and phosphorothioate-modified DNA strands. This method maintained the improved fluorescence efficacy and small size of the QDs while enabling DNA modification. A cholesterol moiety was included at the terminal end of the DNA strand to facilitate specific identification and binding to lipid membranes. This labeling technique attained significant efficiency with minimal effect on viral infectivity, which was effectively utilized to label SARS-CoV-2 spike-pseudotyped enveloped virions. Moreover, the technique had a singular, brief incubation step and could be completed within an hour. By simultaneously labeling viral envelopes with functionalized CdTe QDs and viral nucleic acids with Ru[(phen)_2_(dppz)]^2+^, dual-color fluorescent labeling of SARS-CoV-2 spike-pseudotyped virions is realized. This enabled the real-time visualization and analysis of viral infection dynamics, offering a powerful tool for exploring the underlying mechanisms of viral infection.

## 2. Materials and Methods

### 2.1. Cell Culture

Human embryonic kidney 293T (HEK-293T) cells and African green monkey kidney cells (Vero-E6) were cultivated in Dulbecco’s Modified Eagle Medium (DMEM, Gibco, Thermo Fisher Scientific, Waltham, MA, USA) supplemented with 10% (*v*/*v*) fetal bovine serum (FBS, Gibco, Thermo Fisher Scientific, Waltham, MA, USA) and 1% (*v*/*v*) Penicillin–Streptomycin Solution (100×, comprising 10,000 U/mL penicillin and 10 mg/mL streptomycin, Proteintech Group, Wuhan, China).

### 2.2. Synthesis of Chol-DNA-Functionalized CdTe QDs

Cadmium chloride (CdCl_2_, 113 mg, Sigma-Aldrich, St. Louis, MO, USA) and N-acetylcysteine (NAC, 147 mg, Solarbio, Beijing, China) were weighed and transferred into a 50 mL centrifuge tube. Then, 38 mL of ultrapure water was added and thoroughly agitated until homogeneous. The pH of the resultant solution was further adjusted to 9.0 with 10 M sodium hydroxide (NaOH), producing the Cd-NAC ligand solution. Subsequently, sodium tellurite (Na_2_TeO_3_, 11 mg, Sigma-Aldrich, St. Louis, MO, USA) and sodium borohydride (NaBH_4_, 5 mg, Sigma-Aldrich, St. Louis, MO, USA) were dissolved in 1 mL of ultrapure water to generate separate precursor solutions. While maintaining continuous agitation, these two solutions were gradually introduced dropwise to the previously produced 38 mL Cd-NAC ligand solution, ensuring comprehensive mixing after each addition. The final mixture’s aliquot (2 mL) was used to solubilize the phosphorothioate and chol-modified DNA (sequence: 5′-G*G*G*G*G*G*G*G*G*G*AGTGCTACGA-Cholesteryl-3′, where * denotes a phosphorothioate linkage). The solution was subsequently put into a vial of a Biotage Microwave Reaction Kit (Biotage, Uppsala, Sweden). The synthesis assisted by microwave was conducted utilizing a Biotage Initiator + synthesizer (Biotage, Uppsala, Sweden) at 175 °C for 30 s, resulting in a crude stock solution of Chol-DNA-functionalized QDs. After completing the reaction and cooling to room temperature, the crude product was purified and concentrated using an Amicon Ultra-4 centrifugal filter device (molecular weight cut-off: 50 kDa; Millipore, Burlington, MA, USA) with centrifugation at 6189× *g* for 10 min. The concentration was rinsed three times with ultrapure water to remove residual contaminants, resulting in the final purified Chol-DNA-functionalized QDs. Characterization was performed using transmission electron microscope (TEM; HT7800, Hitachi High-Tech Corporation, Tokyo, Japan), dynamic light scattering (Zetasizer Nano ZSE, Malvern Panalytical, Malvern, UK), and X-ray photoelectron spectroscopy (XPS; K-Alpha, Thermo Fisher Scientific, Waltham, MA, USA).

### 2.3. SARS-CoV-2 Spike-Pseudotyped Virions Production and Purification

HEK-293T cells were cultured in 10 cm culture dishes at a density of 5 × 10^6^ cells per dish for 16 h before transfection. Transfection was conducted utilizing Lipofectamine 8000 reagent (Beyotime Biotechnology, Shanghai, China) in Opti-MEM medium. The envelope-deficient HIV-1 proviral plasmid pNL4-3-KFS (10 µg) and SARS-CoV-2 spike protein expression plasmid pcDNA3.1-S (10 µg) were co-transfected per dish, as per our previous research [31]. The SARS-CoV-2 spike construct used in this study was derived from the severe acute respiratory syndrome coronavirus 2 isolate Wuhan-Hu-1 (GenBank: MN908947.3, https://www.ncbi.nlm.nih.gov/nuccore/MN908947.3, accessed on 15 September 2026). Following 6 h of incubation, the transfection medium was substituted with complete growth medium (DMEM augmented with 10% fetal bovine serum and 1% penicillin-streptomycin). Approximately 18 h post-transfection, the culture supernatant was collected. The supernatant was centrifuged at 238× *g* for 5 min to eliminate cellular debris and filtered using a 0.45 µm membrane filter (Millipore, Burlington, MA, USA). The cleared supernatant was subsequently combined with an equal amount of 20% PEG20000 (Solarbio, Beijing, China) dissolved in 0.9% NaCl solution and incubated at 4 °C for 16 h to facilitate virus precipitation. Following incubation, the mixture was centrifuged at 6791× *g* for 20 min at 4 °C. The supernatant was carefully removed, and the resultant viral pellet was gently resuspended in 1 mL of Opti-MEM medium (Gibco, Thermo Fisher Scientific, Waltham, MA, USA) to provide the concentrated SARS-CoV-2 spike-pseudotyped virions stock solution. Aliquots of the viral stock were made and preserved at −80 °C for subsequent application.

### 2.4. Dual-Color Fluorescent Labeling of SARS-CoV-2 Pseudovirus

The SARS-CoV-2 spike-pseudotyped virions was labeled using separate methodologies for the viral envelope and genome. The viral envelope was labeled with Chol-DNA-functionalized QDs by the interaction between the lipid constituents of the pseudoviral envelope and the cholesterol moiety in the Chol-DNA-functionalized QDs. For labeling, concentrated SARS-CoV-2 spike-pseudotyped virion solution (200 μL) was treated with 1 μL of 30 μM Chol-DNA-functionalized QDs, yielding a final QD concentration of 0.15 μM. The mixture was incubated at 37 °C for 1 h. To facilitate dual-color imaging and confirm successful envelope labeling, the pseudovirus was further labeled with the lipophilic fluorescent dye DiD. To do this, 1 μL of 1 mM DiD was incorporated into the QD-labeled pseudovirus culture to a final concentration of 5 μM, and then incubated at 37 °C for 20 min. The colocalization of green fluorescence from the QDs and red fluorescence from DiD under microscopy was utilized to verify the successful labeling of the pseudoviral envelope.

The nucleic-acid-specific molecular switch Ru[(phen)_2_(dppz)]^2+^ was employed for the labeling of the viral genome. A stock solution of Ru[(phen)_2_(dppz)]^2+^ (10^−4^ mol/L in ultrapure water) was filtered through a 0.45 μm membrane and utilized to treat the virus-producing cells. The filtered working solution (1 mL) was introduced to each 10 cm culture dish at 6 h post-transfection, during which the cells had been transfected with the envelope-deficient HIV-1 proviral pNL4-3-KFS and the S-protein-expressing plasmid pcDNA3.1-S. Incubation continued to facilitate the integration of Ru[(phen)_2_(dppz)]^2+^ into the viral RNA during particle formation. After performing the conventional PEG20000-based concentration and purification procedures, Ru[(phen)_2_(dppz)]^2+^-labeled pseudovirus was acquired.

Dual-color fluorescent pseudoviruses were generated by combining envelope and genome labeling techniques. The purified Ru[(phen)_2_(dppz)]^2+^-labeled pseudovirus (200 μL) was treated with 1 μL of Chol-DNA-functionalized QDs (30 μM) at 37 °C for 1 h, resulting in particles co-labeled with Ru[(phen)_2_(dppz)]^2+^ (genome) and QDs (envelope). For imaging, the co-labeled pseudovirus preparations, comprising either Chol-DNA-functionalized QDs with DiD (envelope double-label) or Chol-DNA-functionalized QDs with Ru[(phen)_2_(dppz)]^2+^ (envelope + genome), were utilized in glass-bottom dishes (Cellvis). Live-cell fluorescence imaging was conducted with excitation wavelengths of 488 nm for Chol-DNA-functionalized QDs and 561 nm for both DiD and Ru[(phen)_2_(dppz)]^2+^, enabling concurrent detection of the fluorescent signals related to the viral envelope and genome.

### 2.5. Western Blotting

Protein expression in SARS-CoV-2 spike-pseudotyped virion samples were evaluated using Western blotting. Cell and virus samples were lysed on ice for 30 min in RIPA buffer (Thermo Fisher Scientific, Waltham, MA, USA) supplemented with a protease inhibitor cocktail (Beyotime Biotechnology, Shanghai, China) and centrifuged at 13,523× *g* for 15 min at 4 °C to collect the supernatants, which were then denatured at 95 °C for 5 min. Equal quantities of protein were added and separated using 10% SDS-PAGE, initially at 80 V and subsequently at 120 V in the resolving gel. Proteins were transferred to PVDF membranes (Millipore, Burlington, MA, USA) using wet transfer at 300 mA and 4 °C, with transfer times optimized for the molecular weights of the target proteins. Membranes were incubated with 5% non-fat milk in TBST for 1 h at room temperature, treated overnight at 4 °C with primary antibodies against, including rabbit anti-SARS-CoV-2 S (1:1000, Sino Biological, Beijing, China), mouse anti-SARS-CoV-2 S1 (1:2000; Sino Biological), mouse anti-p24 (1:2000, Abcam, Cambridge, UK), or mouse anti-GAPDH (1:2000, Cell Signaling Technology, Danvers, MA, USA), washed with TBST, and probed with HRP-conjugated secondary antibodies (anti-rabbit or anti-mouse IgG, 1:2000; Cell Signaling Technology) for 1 h at room temperature. Protein bands were visualized via an enhanced chemiluminescence detection kit (Beyotime Biotechnology, Shanghai, China), and detection signals were recorded with a ChemiDoc MP system (Bio-Rad, Hercules, CA, USA).

### 2.6. TEM Imaging

The SARS-CoV-2 spike-pseudotyped virion suspension (10 μL) was placed on a carbon-coated copper grid and allowed to adhere for 1 min at room temperature. Excessive liquid was eliminated by blotting with filter paper (Millipore, Burlington, MA, USA). The grid was subsequently negatively stained with 10 μL of a 2% phosphotungstic acid solution (Solarbio, Beijing, China) at pH 6.5 for 1 min, after which excess stain was removed using filter paper (Millipore, Burlington, MA, USA). The grid was air-dried at room temperature for several minutes. Images were obtained using a TEM at 80–120 kV for further investigation.

### 2.7. Virus Infection

Vero-E6 cells (5 × 10^4^ cells/dish) were cultured in glass-bottomed dishes for 16 h before imaging, to facilitate single-particle tracking of fluorescently labeled viral particles. The co-labeled fluorescent particles were incubated with the cells for 20 min, and then washed with fresh media to eliminate unattached particles. Live-cell imaging was conducted utilizing an UltraView Vox spinning-disk confocal laser scanning system (Perkin Elmer, Waltham, MA, USA) mounted to a Nikon Ti-e inverted microscope (Nikon Corporation, Tokyo, Japan), with a 60× oil immersion objective attached. Images were continually captured for 15 min at 10 s intervals at 37 °C and 5% CO_2_. The resultant time-lapse image stacks were processed with Imaris 10.0.0 software (Bitplane, Belfast, UK) for tracking particle trajectories and performing mean square displacement (MSD) analysis.

### 2.8. Real-Time PCR

To evaluate whether QD labeling alters viral infectivity, Vero-E6 cells were separately infected with QD-modified virus and native virus. For the inhibition control group, cells were preincubated with 10 μmol/L nevirapine (Sigma-Aldrich, St. Louis, MO, USA) for 1 h prior to pseudovirus infection, with nevirapine retained in culture medium throughout the incubation period. After 2 h of viral adsorption followed by 24 h incubation, total cellular RNA was extracted and reverse-transcribed into cDNA. Intracellular HIV-1 p24 gene levels were quantified by qPCR (Hangzhou Bioer Technology, Hangzhou, China). All experiments were performed in triplicate, and the results are presented as mean ± SD.

### 2.9. Cell Viability Assay of Chol-DNA-Functionalized QDs via CCK-8

Vero-E6 and 293T cells (1.5 × 10^3^/well) were seeded in 96-well plates (Corning, Corning, NY, USA) and cultured overnight at 37 °C, 5% CO_2_. Cells were treated with serially diluted Chol-DNA-functionalized QDs (0–200 nM) for 24 h, followed by CCK-8 (10 µL/well, MedChemExpress, Monmouth Junction, NJ, USA) incubation for 2 h. Absorbance was measured on a TECAN microplate reader (Tecan Group, Männedorf, Switzerland). Cell viability was calculated as [(As − Ab)/(Ac − Ab)] × 100%, where As, Ac, and Ab denote experimental, control, and blank wells, respectively. Each concentration was tested in *n* = 6 biological replicates; data are presented as mean ± SD.

### 2.10. Image Analysis

Imaris software was employed for confocal image data of three-dimensional reconstruction, rendering, and viral particle tracking. Furthermore, Image J 1.54r (NIH, Bethesda, MD, USA) was utilized for the quantification of fluorescence intensity, area, and particle counts.

### 2.11. Statistical Analysis

Quantitative data are expressed as mean ± standard deviation (SD). Statistical analysis was performed using GraphPad Prism software 9.5.1 (GraphPad Software, San Diego, CA, USA).

## 3. Results and Discussion

### 3.1. Preparation and Characterization of Chol-DNA-Functionalized QDs

**Figure 1:**
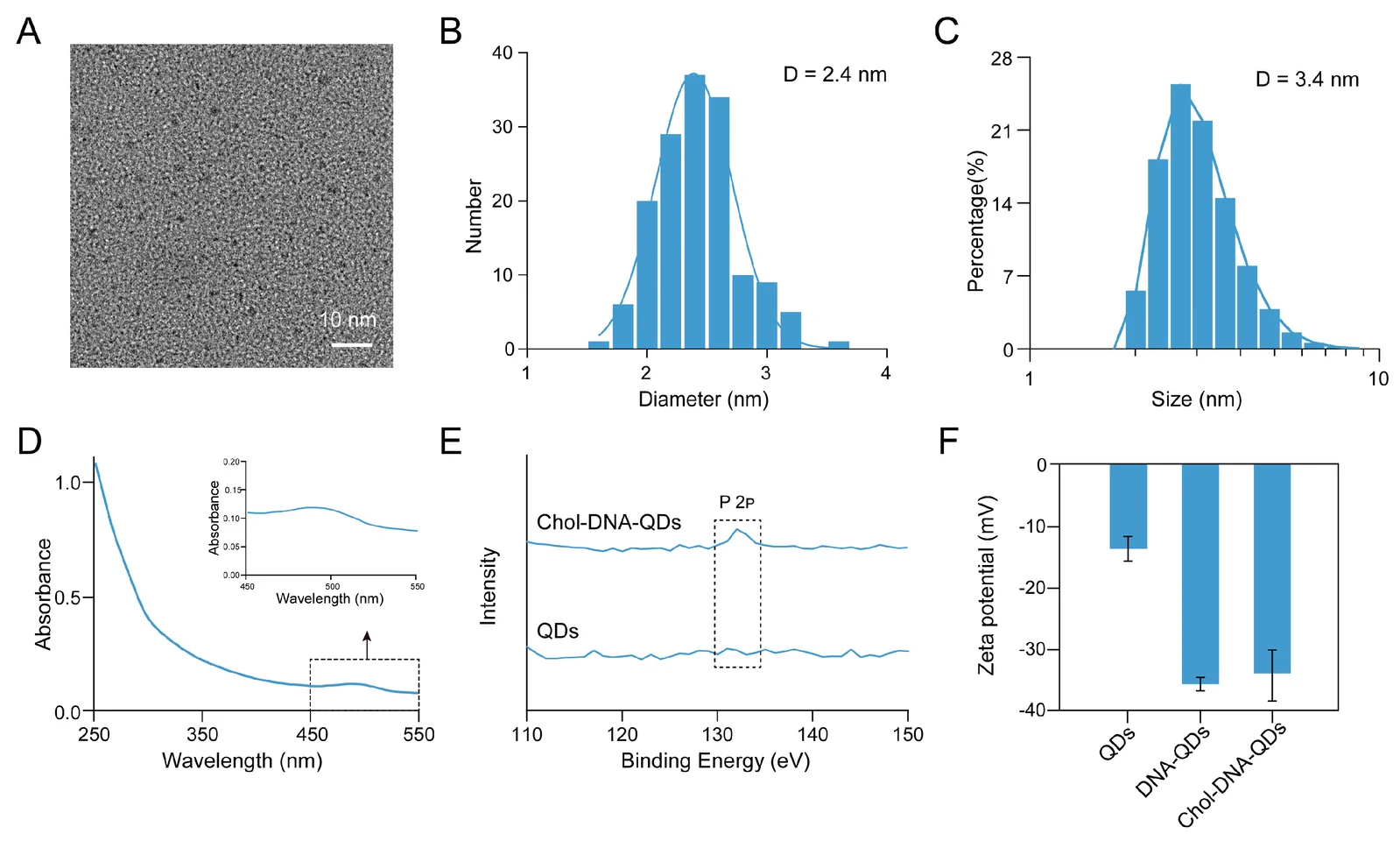
Preparation and characterization of Chol-DNA-functionalized CdTe QDs. (**A**) TEM imaging of Chol-DNA-functionalized QDs. Scale bar: 20 nm. (**B**) Size and (**C**) DLS distribution of Chol-DNA-functionalized QDs. (**D**) UV–vis absorption spectra of Chol-DNA-functionalized QDs. (**E**) XPS spectra of P2p for Chol-DNA-functionalized QDs and unmodified QDs. (**F**) Zeta potential of QDs, DNA-functionalized QDs, and Chol-DNA-functionalized QDs.

The size and morphology of the Chol-DNA-functionalized CdTe QDs were assessed using transmission electron microscopy (TEM). Figure 1A demonstrates a spherical and uniform distribution of the Chol-DNA-functionalized QDs. The measurement of 152 randomly selected particles indicated an average diameter of 2.4 nm (Figure 1B). The hydrodynamic diameter was determined to be 3.41 nm using dynamic light scattering (Figure 1C). The UV–vis absorption spectra in Figure 1D exhibit a prominent peak at 490 nm, aligning with the typical absorption of CdTe QDs. The Chol-DNA-functionalized QDs exhibit a distinct fluorescence peak at 545 nm under varying excitation wavelengths (Figure S1). To further confirm conjugation, unmodified and Chol-DNA-functionalized QDs were examined using XPS. Figure 1E illustrates the appearance of distinct peaks associated with the P2p orbital in the XPS spectra of Chol-DNA-functionalized QDs, confirming the presence of the conjugated DNA and suggesting successful alteration of the QDs. Furthermore, zeta potential measurements (Figure 1F) indicate values of −13.9 mV, −35.9 mV, and −34.2 mV for QDs, DNA-functionalized QDs, and Chol-DNA-functionalized QDs, respectively. In comparison to unmodified QDs, the zeta potential of Chol-DNA-functionalized QDs exhibited significant alteration towards a more negative value. No substantial distinction was observed between DNA-functionalized QDs and Chol-DNA-functionalized QDs, suggesting that the cholesterol moiety did not substantially affect the charge properties of the DNA. These results collectively validate the effective production of Chol-DNA-functionalized QDs. Furthermore, cell viability assays demonstrated that Chol-DNA-functionalized QDs treatment exerted negligible cytotoxic effects on both Vero-E6 and 293T cells, indicating the excellent biocompatibility of the developed labeling probe (Figure S2).

### 3.2. Construction and Characterization of Virus Particles

**Figure 2:**
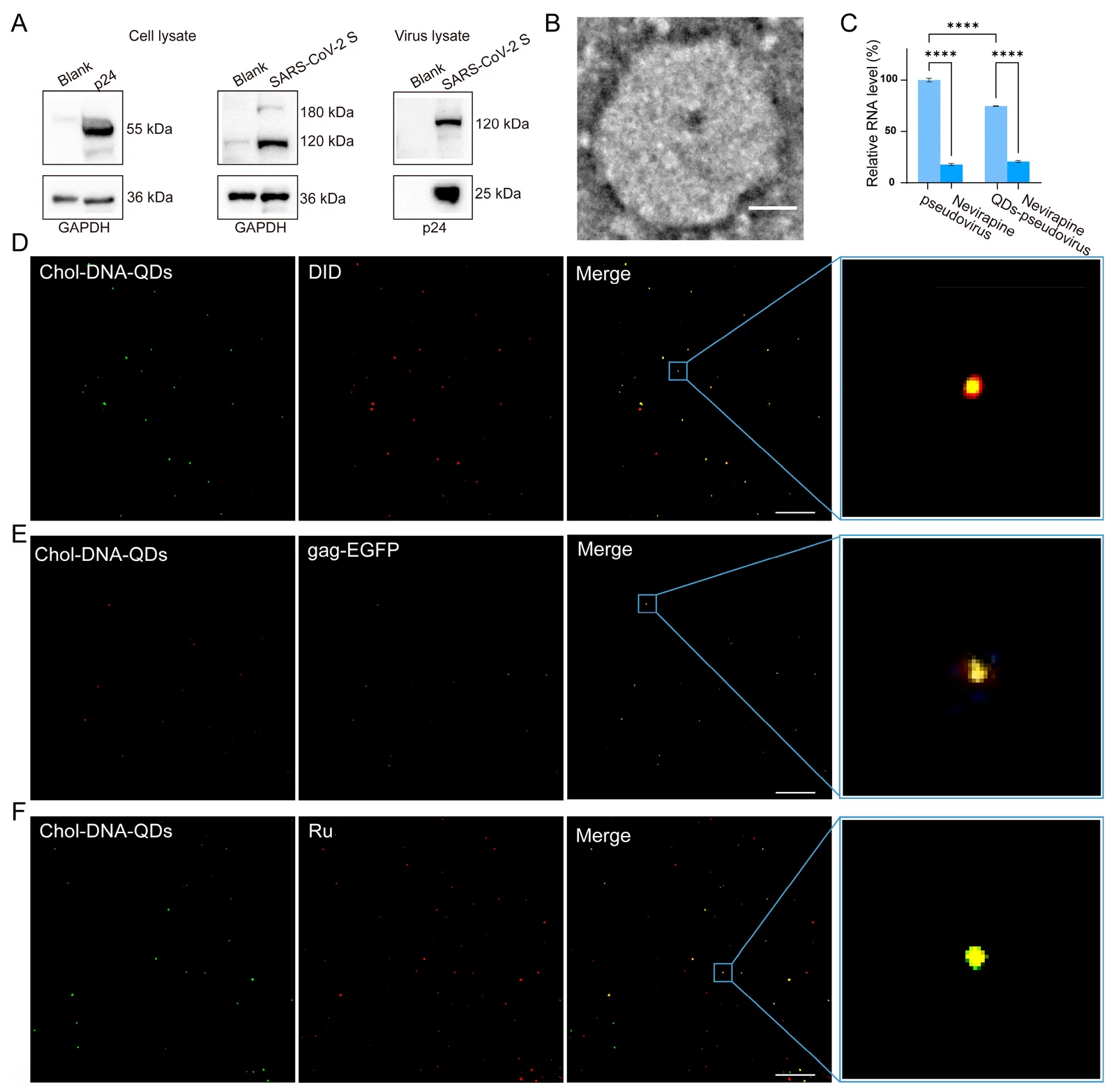
Construction and characterization of labeled pseudovirus particles. (**A**) Western blot assessment of S and p24 proteins in cell lysates and pseudoviruses. (**B**) TEM imaging of SARS-CoV-2 spike-pseudotyped virions. Scale bar: 50 nm. (**C**) RT-qPCR analysis of relative RNA levels of pseudoviruses with or without Chol-DNA-functionalized QDs labeling in the presence or absence of nevirapine. **** indicates *p* value < 0.0001. (**D**) Colocalization analysis of Chol-DNA-functionalized QDs and DiD labeling. Scale bar: 30 μm. (**E**) Colocalization analysis of gag-EGFP and Chol-DNA-functionalized QDs. Scale bar: 30 μm. (**F**) Colocalization analysis of Chol-DNA-functionalized QDs and Ru[(phen)_2_(dppz)]^2+^ labeling. Scale bar: 30 μm.

We then prepared SARS-CoV-2 spike-pseudotyped virions by co-transfecting 293T cells with plasmids that encode the SARS-CoV-2 spike protein and HIV-1 Gag-Pol, resulting in envelope-deficient virus-like particles. Immunoblotting analysis of the produced cell lysates validated the production of the HIV-1 Gag precursor (p55, identified through p24), the complete SARS-CoV-2 S protein (~190 kDa), and its S1 subunit (~110 kDa) (Figure 2A). Distinct bands for both p24 and the S protein were observed in the cell lysate and pure virus, signifying the successful assembly and collection of pseudoviral particles (Figure 2A). The TEM examination of the pure virus particles revealed an average diameter of about 100 nm. Distinct S-like projections, approximately 10 nm in length, were observed on the surface of these particles, validating the morphogenesis of SARS-CoV-2 spike-pseudotyped virions exhibiting the SARS-CoV-2 S protein (Figure 2B and Figure S3). To assess the effect of fluorescent labeling on viral infectivity, the relative RNA levels of infectious viruses were evaluated using RT-PCR. The findings demonstrate that following the labeling of the viral envelope with Chol-DNA-functionalized QDs via the interaction between viral lipids and cholesterol in Chol-DNA-functionalized QDs, the relative RNA level of the virus was maintained at 86.7% compared to the unlabeled virus. Treatment with nevirapine, a non-nucleoside reverse-transcriptase inhibitor of HIV-1, effectively suppressed viral replication in infected cells, indicating the high specificity of the RT-PCR assay employed in our experiments. The data demonstrated that the QD labeling method had no substantial impact on viral infectivity (Figure 2C). Subsequently, the viruses were labeled using Chol-DNA-functionalized QDs and Ru[(phen)_2_(dppz)]^2+^, respectively. Confocal images showed the absence of fluorescence crosstalk among the various channels, and the dual labeling exhibited no interference with future tests (Figures S4 and S5).

A colocalization test was conducted using the lipophilic membrane dye DiD to validate the selectivity of Chol-DNA-functionalized QDs for the viral envelope. The images indicate that almost 89.6% of the fluorescence signals from Chol-DNA-functionalized QDs co-localized with those of DiD, illustrating the high selectivity of QD labeling for the viral membrane (Figure 2D). To further verify the colocalization of cholesterol-QD conjugates with viral components, we conducted colocalization assays using the virus-specific Gag-EGFP marker. The results showed that Chol-DNA-functionalized QDs fluorescence signals substantially overlapped with Gag-EGFP signals, with a colocalization efficiency of 96.95%. This confirms that our labeling strategy can efficiently target HIV-1 viral particles (Figure 2E). For rapid and continuous viral monitoring over prolonged periods, a dual-labeled pseudovirus was developed utilizing Chol-DNA-functionalized QDs and Ru[(phen)_2_(dppz)]^2+^. During synthesis, the viral RNA genome was intercalated with the fluorescent probe Ru[(phen_)2_(dppz)]^2+^. Subsequently, the envelope of the extracted virions was labeled with Chol-DNA-functionalized QDs. Fluorescence imaging demonstrated that around 67.8% of the Chol-DNA-functionalized QD-labeled particles displayed colocalization with the genomic signal from Ru[(phen)_2_(dppz)]^2+^ (Figure 2F). The observed partial colocalization is mainly attributed to spontaneous premature uncoating and partial genome release of a fraction of virions during staining, which diminishes genomic RNA signals. In addition, uneven loading of the Ru[(phen_)2_(dppz)]^2+^ probe among individual viral particles also restricted colocalization efficiency. Collectively, these results validate that Chol-DNA-functionalized QDs can proficiently label the viral envelope, regardless of an internally labeled genome.

### 3.3. Live-Cell Imaging of SARS-CoV-2 Spike-Pseudotyped Viral Entry

To assess the efficacy of the labeling technique, SARS-CoV-2 spike-pseudotyped virions, dually labeled with Ru[(phen)_2_(dppz)]^2+^ and Chol-DNA-functionalized QDs, were employed to infect Vero-E6 host cells. In the merged channel, the dual-labeled SARS-CoV-2 spike-pseudotyped virions manifested as yellow dots (Figure S6A). Time-lapse imaging revealed that the virus initially colocalized with the host cell. Following a brief adhesion, the dissociation of the viral envelope from the genome was captured, as demonstrated by the disappearance of the red fluorescent-labeled genome from the green fluorescent-labeled viral envelope (Figure S6B, Movie S1). MSD analysis further confirmed that the envelope underwent active transport after genome release, as reflected by the upward-curved MSD profile (Figure S6C). These results demonstrate that our labeling technique enables effective tracking and analysis of viral infection dynamics.

## 4. Conclusions

In this work, we established a Chol-DNA-functionalized QDs labeling method for the simple and efficient labeling of enveloped viruses. Utilizing the natural affinity between viral lipid envelopes and QD-tethered cholesterol, targeted fluorescent labeling is accomplished via one short incubation within approximately one hour, with viral structure and infectivity fully maintained. The one-pot synthesized CdTe QDs derived from Cd^2+^ and phosphorothioate-modified DNA feature bright fluorescence, small particle size and easy DNA functionalization. Moreover, cytotoxicity validation confirmed the good biocompatibility of Chol-DNA-functionalized QDs, further supporting its reliable application in viral imaging. Integrated with Ru[(phen)_2_(dppz)]^2+^ nucleic acid staining, this lipid-specific labeling strategy enables dual-color fluorescence imaging of SARS-CoV-2 spike-pseudotyped virions, supporting the real-time tracking of viral infection dynamics. This robust and versatile QD labeling approach compensates for the limitations of conventional viral labeling techniques, providing a powerful platform for single-virus imaging, precise viral detection, and in-depth mechanistic investigation of virus infection.

## Data Availability

The original contributions presented in this study are included in the article. Further inquiries can be directed to the corresponding author.

## Supplementary Materials

The following supporting information can be downloaded at https://www.mdpi.com/article/10.3390/v18101072/s1, Figure S1: Fluorescence spectra under different excitation wavelengths; Figure S2: Cell viabilities of Vero-E6 (A) and 293T (B) cells after Chol-DNA functionalized QDs treatment; Figure S3: Statistical quantification of protein distribution in each ultrathin section; Figure S4: Confocal image of Chol-DNA-functionalized QDs labeled SARS-CoV-2 spike-pseudotyped virions; Figure S5: Confocal image of Ru[(phen)_2_(dppz)]^2+^ labeled SARS-CoV-2 spike-pseudotyped virions; Figure S6: Live-cell imaging of SARS-CoV-2 spike-pseudotyped virions entry; Movie S1: Real-time imaging of SARS-CoV-2 spike-pseudotyped virions entry into Vero-E6 cells.
