# Supplementary material for: Single-Particle Tracking via Direct Labeling of the Viral Lipid Envelope with Cholesterol-DNA-Functionalized Quantum Dots
Source: Viruses. 2026 Sep 28;18(10):1072. doi: 10.3390/v18101072 (PMC13709940; doi:10.3390/v18101072)
Supplement: Supplementary file 1 [file viruses-18-01072-s001.zip › viruses-4557468-supplementary.pdf]

Supporting Information

Single-particle tracking via direct labeling of the viral lipid envelope with Cholesterol-DNA-functionalized quantum dots

Wen Yin[1, #], Xiaoxu Guo[1, 2, #], Jie Zhou[1,2], Guobin Mao[2,*], Yingxin Ma[2,*]

[1]State Key Laboratory of Biocatalysis and Enzyme Engineering, Hubei Key Laboratory of Industrial Biotechnology, School of Life Sciences, Hubei University, Wuhan 430062, China

[2]Materials Artificial Intelligence Center, Shenzhen Institute of Advanced Technology, Chinese Academy of Sciences, Shenzhen 518055, China

[*]Correspondence: Guobin Mao (gb.mao@siat.ac.cn) Yingxin Ma (yx.ma1@siat.ac.cn)

[#]Wen Yin, and Xiaoxu Guo contributed equally to this work.

Table of Contents

Figure S1. Fluorescence spectra under different excitation wavelengths.

Figure S2. Cell viabilities of Vero-E6 (A) and 293T (B) cells after Chol-DNA functionalized QDs treatment.

Figure S3. Statistical quantification of protein distribution in each ultrathin section.

Figure S4. Confocal image of Chol-DNA functionalized QDs labeled SARS-CoV-2 spike-pseudotyped virions.

Figure S5. Confocal image of $Ru[(phen)_2(dppz)]^{2+}$ labeled SARS-CoV-2 spike-pseudotyped virions.

Figure S6. Live-cell imaging of SARS-CoV-2 spike-pseudotyped virions entry.

Movie S1. Real-time imaging of SARS-CoV-2 spike-pseudotyped virions entry into Vero-E6 cells.

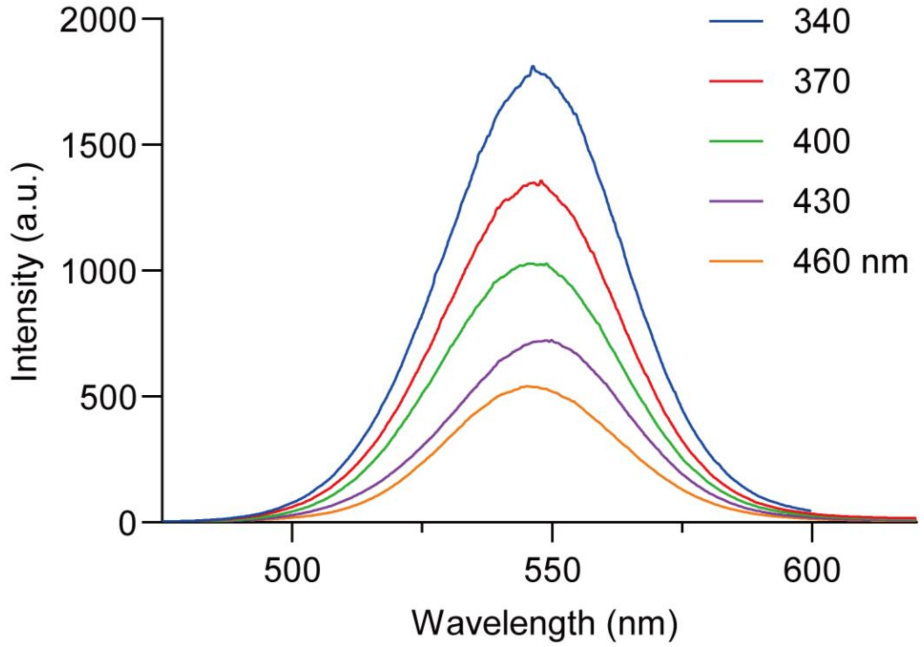

Figure S1. Fluorescence spectra under different excitation wavelengths.

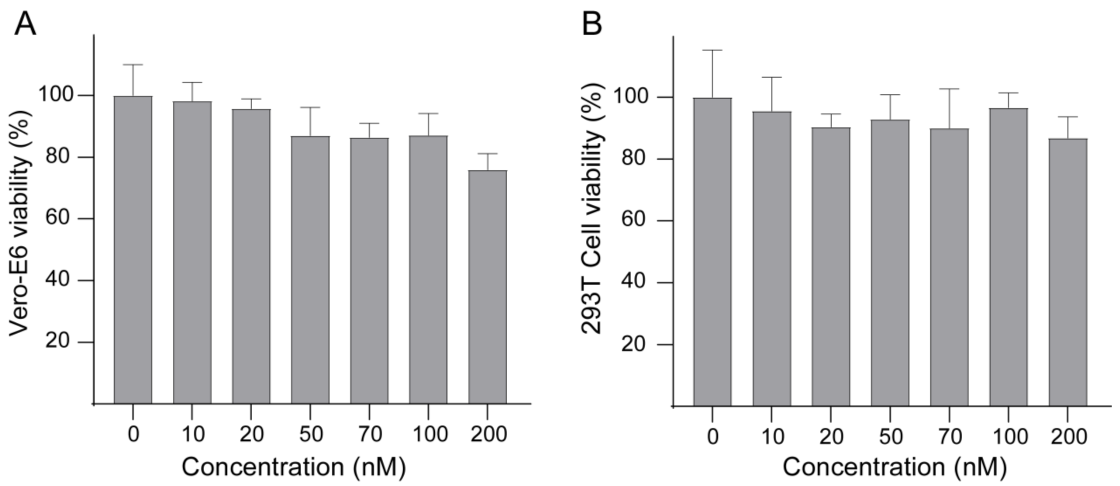

Figure S2. Cell viabilities of Vero-E6 (A) and 293T (B) cells after Chol-DNA functionalized QDs treatment.

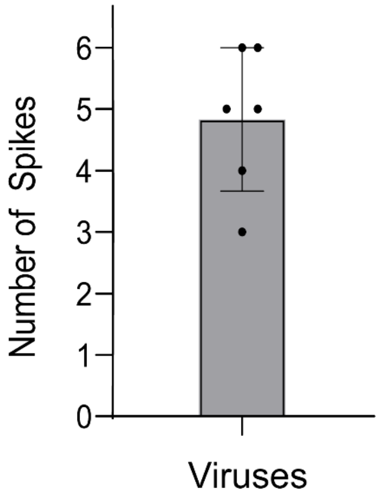

Figure S3. Statistical quantification of protein distribution in each ultrathin section.

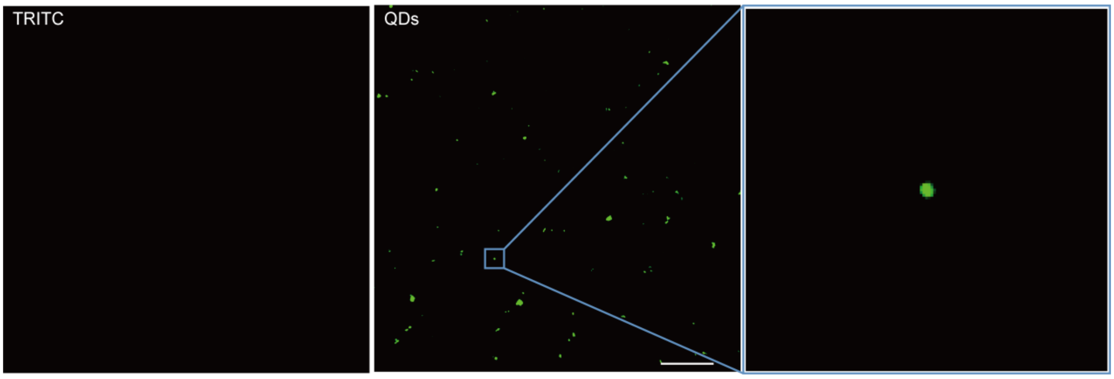

Figure S4. Confocal image of Chol-DNA functionalized QDs labeled SARS-CoV-2 spike-pseudotyped virions. Scale bar: 30 μm.

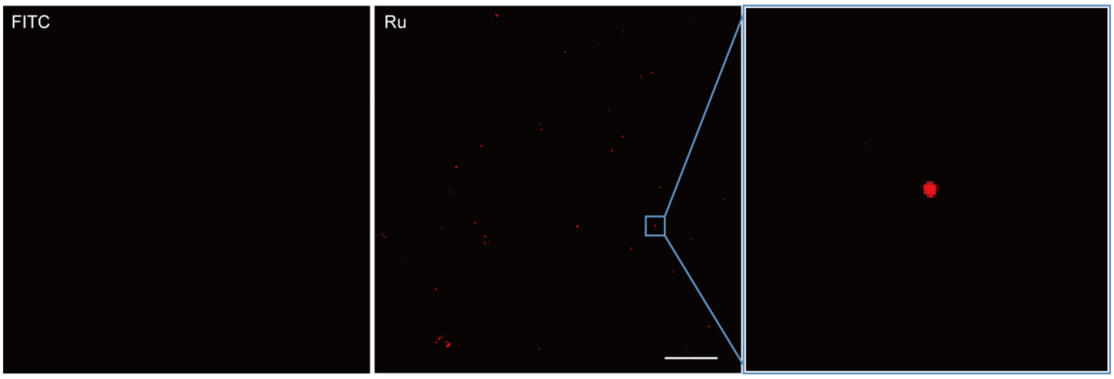

Figure S5. Confocal image of $Ru[(phen)_2(dppz)]^{2+}$ labeled SARS-CoV-2 spike-pseudotyped virions. Scale bar: 30 μm.

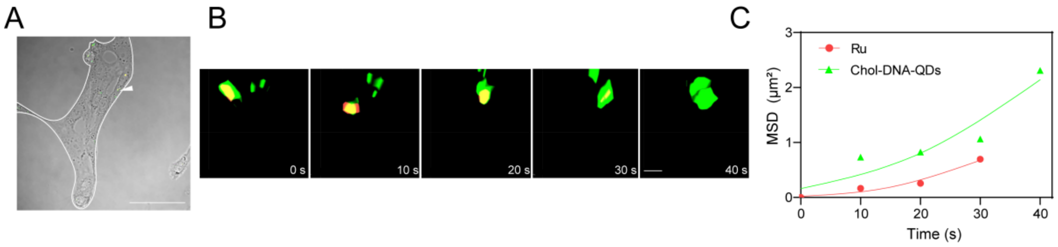

Figure S6. Live-cell imaging of SARS-CoV-2 spike-pseudotyped virions entry. (A) Brightfield image of host cells with dual-labeled virions. Scale bar: 100 μm. (B) Time-lapse fluorescence images showing the spatial separation of the Chol-DNA-functionalized QDs labeled envelope and $Ru[(phen)_2(dppz)]^{2+}$ labeled viral genome. Scale bar: 1 μm. (C) MSD analysis of Chol-DNA-functionalized QDs labeled envelope and $Ru[(phen)_2(dppz)]^{2+}$ labeled viral genome.

Movie S1 Real-time imaging of SARS-CoV-2 spike-pseudotyped virions entry into Vero-E6 cells.
